# Determination of d- and l-Amino Acids in Garlic Foodstuffs by Liquid Chromatography–Tandem Mass Spectrometry

**DOI:** 10.3390/molecules28041773

**Published:** 2023-02-13

**Authors:** Mayu Onozato, Haruna Nakanoue, Tatsuya Sakamoto, Maho Umino, Takeshi Fukushima

**Affiliations:** Department of Analytical Chemistry, Faculty of Pharmaceutical Sciences, Toho University, 2-2-1 Miyama, Funabashi-shi 274-8510, Japan

**Keywords:** black garlic, fermentation, d-serine, d-aspartic acid, CIMa-OSu, chiral derivatization, LC–MS/MS

## Abstract

Black garlic is currently attracting interest as a health food and constituent of commercial supplements; however, no data regarding the d-amino acids within black garlic have been reported. Therefore, the amino acid compositions of methanol extracts from fresh and black garlic were compared herein. We investigated the contents of the d- and l-forms of amino acids in commercial fresh, black, and freeze-dried garlic foodstuffs by liquid chromatography–tandem mass spectrometry (LC–MS/MS) using a pre-column chiral derivatization reagent, succinimidyl 2-(3-((benzyloxy)carbonyl)-1-methyl-5-oxoimidazolidin-4-yl) acetate. Several d-amino acids, namely, the d-forms of Asn, Ala, Ser, Thr, Glu, Asp, Pro, Arg, Phe, Orn, Lys, and Tyr, were observed in the methanol extract of black garlic, whereas only d-Ala was detected in that of fresh garlic foodstuffs. These data suggest that several d-amino acids can be produced during fermentation for preparing black garlic.

## 1. Introduction

Edible garlic is used as a spice in the cuisines of most countries and has also attracted attention as a medicinal ingredient. As a foodstuff, fresh garlic (FG), *Allium sativum* L., is generally eaten as a raw grated vegetable, whereas black garlic (BG) is a health-oriented foodstuff that is prepared from FG by steaming in a rice cooker under humid conditions at approximately 60–90 °C for 14–30 days [1]. BG is used as a daily nutritional supplement [2]. Garlic fermentation occurs during the preparation of BG from FG, which causes significant changes in its constituents [3]. Several previous metabolomics studies have indicated that changes in lipids, amino and organic acids, sulfur-containing compounds, and sugar occur as functions of the fermentation period [3]. The pharmacological efficacies of BG against several diseases, such as hypertension, atherosclerosis, diabetes, cancer, and neurodegenerative diseases, have been investigated [1].

In terms of critical constituents, FG contains several sulfur-containing compounds, namely, allicin, *S*-allyl-l-cysteine (SAC), and *γ*-glutaryl-*S*-allyl-l-cysteine [2]. SAC exhibits anti-allergic [1] and anti-hypertensive [3] bioactivities, offers protection against diabetes [2], and induces enhanced immune-cell functions. However, while fermentation progresses, the SAC levels remarkably decrease in BG [3,4]. Alterations in the levels of other proteinogenic amino acids as FG becomes BG by steaming have previously been investigated. Molina-Calle et al. have reported increased levels of Ala, Asp, Cys, His, Ile, Leu, Phe, and Pro, as well as decreased levels of Arg, Asp, Cit, Gln, Glu, Lys, and Trp in BG compared with those in FG [4].

In the last two decades, only a few studies have reported on the d-amino acids within garlic, which are found in foods, fruits, and drinks; fermented foods or drinks were reported to typically contain several free forms of d-amino acids [5,6,7,8]. In 1994, Bruckner and Westhauser reported the presence of d-amino acids such as d-Ala, d-Asn, d-Glu, d-Leu, and d-Val in FG [9], but there are no data on the free d-amino acids in BG.

Compared to free l-amino acids, d-amino acids may affect the taste of foodstuffs [10] and body-weight changes [11] differently. In an animal experiment using rats, a daily diet containing 0.3% d-Trp, not l-Trp, was found to cause a loss of body weight gain [11], suggesting that careful attention should be focused on d-Trp consumption. Meanwhile, the efficiency of d-Trp utilization for growth in humans is poor, and humans utilize minimal d-Trp [12]. Thus, the nutritional influences of d-amino acids on daily life should be considered. Recently, some d-amino acids have been found to participate in physiological functions. For example, d-Ser affects a synaptic modulator for cognition through *N*-methyl-d-aspartate (NMDA) receptor, and d-Arg exerts a normalizing effect on glucocorticoid-induced neurotoxic action [13,14]. Therefore, an analysis of such d-amino acids in daily food is needed.

Therefore, data regarding the free d-amino acids in BG as a foodstuff are required. For example, patients with decreased concentrations of d-amino acids are speculated to be able to improve their biological balance by consuming foods containing high levels of these d-amino acids on a daily basis.

To investigate the free-amino acid contents in garlic samples, several recent reports have used instrumental analyses. Using a combination of comprehensive nuclear magnetic resonance (NMR) and multivariate analyses, Liang et al. [15] reported 38 component changes during the process of converting FG to BG. Although the NMR method has an advantage in terms of the easy pretreatment of the garlic sample without requiring a process for separating components, the optical isomers of amino acids are not resolved. Meanwhile, chromatographic techniques such as gas chromatography (GC) [9,16,17] and high-performance liquid chromatography (HPLC) have been widely employed in investigating the free amino acid contents in food samples [17,18,19,20,21,22,23,24].

The application of tandem mass spectrometry (MS/MS) in detection may provide high selectivity in analyzing crude food samples [25,26]. However, to determine the contents of both d- and l-amino acids separately, the use of a chiral stationary phase or diastereomer derivatization reagent is necessary [27]. We have previously developed a method for determining the free d- and l-amino acid contents in a sample of miso using LC–MS/MS with a reversed-phase C8 column and a pre-column diastereomer derivatization reagent, succinimidyl 2-(3-((benzyloxy)carbonyl)-1-methyl-5-oxoimidazolidin-4-yl)acetate (CIMa-OSu) [28]. Considering that partially incomplete separation of the d- and l-amino acids was observed when using the C8 column, in this study, we explored the use of a mixed phase (Scherzo SS-C18^®^) comprising reversed and ionic phases as a separation column. Accordingly, we investigated the d- and l-forms of free amino acids in commercial FG and BG foodstuffs using this improved LC–MS/MS method.

## 2. Results and Discussion

### 2.1. Chromatographic Separation of d- and l-Amino Acids

In our previous study, we used the pre-column chiral derivatization reagent CIMa-OSu and reported that 18 kinds of d- and l-amino acids derivatized with CIMa-OSu were enantiomerically separated on a reversed-phase C8 column via LC–MS/MS.

The d- and l-amino acids derivatized with CIMa-OSu exhibited one or two ionic carboxyl groups. Therefore, a mixed-phase column (Scherzo SS-C18^®^) comprising reversed and ionic phases was used because hydrophobic and ion-exchange interactions occur simultaneously on the stationary phase with analytes bearing ionic groups, such as carboxyl or amino moieties [29].

To select the mobile phase, we investigated several pH-buffered aqueous solutions mixed with H_2_O/MeOH (1/1, *v*/*v*). The use of a buffered solution at pH 2.8 (H_2_O/MeOH = 1/1, *v*/*v*) afforded the excellent separation of most enantiomers of amino acids. The retention times of CIMa-dl-Ser (*m*/*z* 380.10 > 91.10) and CIMa-γ-aminobutyric acid (GABA, *m*/*z* 378.05 > 91.15) (Table S1) varied with the pH (Figure S1a–f, H_2_O/MeOH = 1/1, *v*/*v*). As shown in Figure S1a–g, a small peak originating from the isotopic ion (*m*/*z* 380.1) of CIMa-GABA was detected in the multiple-reaction monitoring (MRM) chromatograms of CIMa-d- and -l-Ser. At pH 2.8 (H_2_O/MeOH = 1/1, *v*/*v*), the peak representing CIMa-GABA overlapped with that of the retention time of CIMa-l-Ser (Figure S1d); thus, the small peak originating from the isotopic ion of CIMa-GABA was included in the peak of CIMa-l-Ser. Under the mobile phase condition, l-Ser cannot be accurately determined.

**Figure 1:**
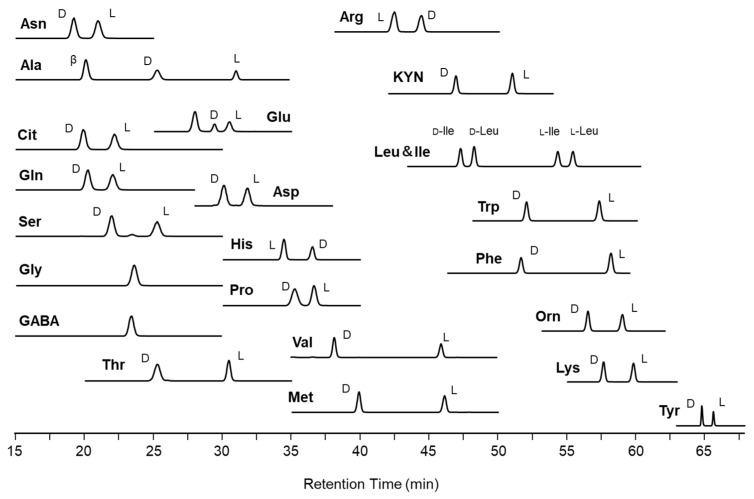
Representative chromatograms of mixed standard d- and l-amino acid samples obtained using the mixed-phase column. The mobile phase conditions are described in the text, Section 3.4.

Thus, the composition of H_2_O/MeOH was optimized, and a good separation of CIMa-d-Ser, -l-Ser, and -GABA was observed at pH 2.8 (H_2_O/MeOH = 5/2, *v*/*v*, Figure S1g). As shown in Table 1, relatively large values of *R*_s_ were obtained using the pH 2.8 buffered solution (H_2_O/MeOH = 5/2, *v*/*v*) as a mobile phase. Representative chromatograms of the amino acid mixtures are shown in Figure 1. Therefore, the use of the mixed-mode column provides an analytical advantage in achieving efficient enantiomeric separations of CIMa-amino acids.

As shown in Table S2, the limit of detection (LOD) of l-Gln was significantly improved over those obtained in the previous method, while those of other amino acids were approximately comparable; the successful lowering of the LODs of d-Ala and d-Ser, which are among the d-amino acids that are more often detected in samples, is expected for future applications to other samples (e.g., biological samples such as human serum).

### 2.2. Analyses of Commercial Garlic Foodstuffs

Using the proposed LC–MS/MS conditions, three types of commercial garlic foodstuffs were analyzed. The calibration curves obtained in the present study showed good linearity (*R*^2^ = 0.999). The intra- and inter-day accuracy and precision were sufficient to determine the contents of free d- and l-amino acids in the garlic foodstuffs (Table S3).

**Figure 2:**
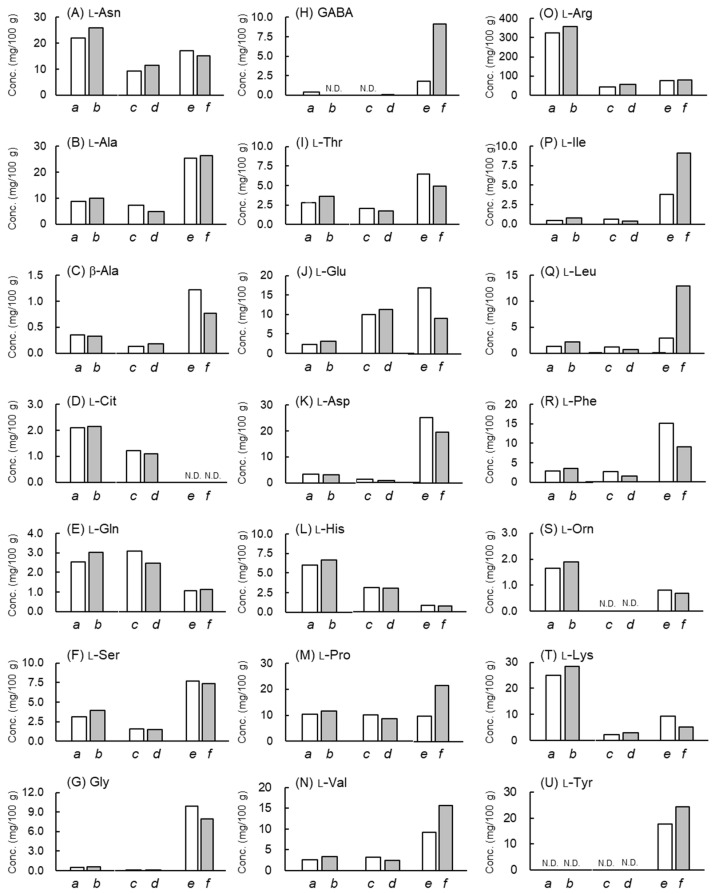
Concentrations (mg/100 g) of free l-amino acids in samples of grated raw garlic (*a*, *b*), freeze-dried garlic (*c*, *d*), and fermented BG (*e*, *f*), as determined in duplicate. (**A**) l-Asn, (**B**) l-Ala, (**C**) β-Ala, (**D**) l-Cit, (**E**) l-Gln, (**F**) l-Ser, (**G**) Gly, (**H**) GABA, (**I**) l-Thr, (**J**) l-Gln, (**K**) l-Asp, (**L**) l-His, (**M**) l-Pro (**N**) l-Val, (**O**) l-Arg, (**P**) l-Ile, (**Q**) l-Leu, (**R**) l-Phe, (**S**) l-Orn, (**T**) l-Lys, (**U**) l-Tyr. N.D.: not detected.

Figure 2 shows the concentrations (mg/100 g) of free l-amino acids in the MeOH extract samples of grated raw garlic, freeze-dried garlic, and fermented BG. Among the free amino acids, l-Arg is present at the highest concentrations in all garlic foodstuffs evaluated in the present study; this extremely high content of l-Arg in FG is consistent with previous results [30,31]. In addition, the concentrations of l-Arg, l-Asn, and l-Lys are the highest in grated raw garlic, whereas those of l-Asp, l-Ala, and l-Phe are the highest in fermented BG.

**Figure 3:**
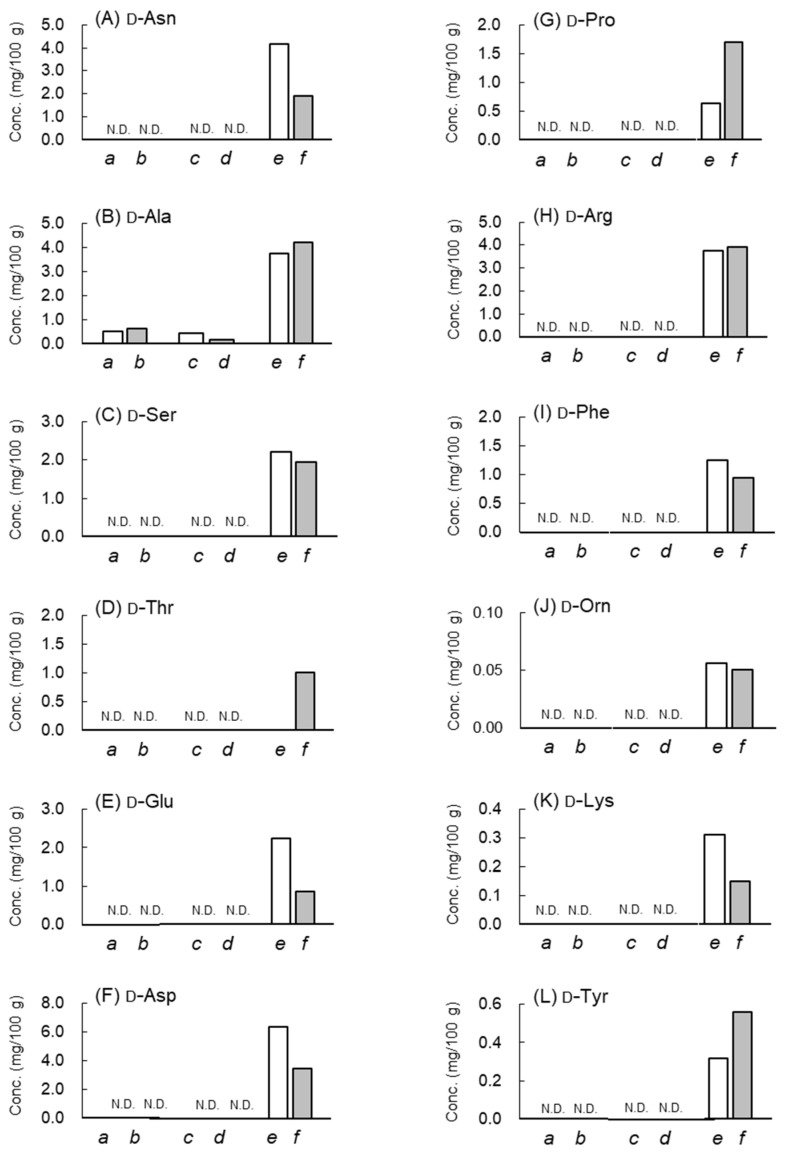
Concentrations (mg/100 g) of free d-amino acids in samples of grated raw garlic (*a*, *b*), freeze-dried garlic (*c*, *d*), and fermented BG (*e*, *f*), as determined in duplicate. (**A**) d-Asn, (**B**) d-Ala, (**C**) d-Ser, (**D**) d-Thr, (**E**) d-Glu, (**F**) d-Asp, (**G**) d-Pro (**H**) d-Arg, (**I**) d-Phe, (**J**) d-Orn, (**K**) d-Lys, (**L**) d-Tyr. N.D.: not detected.

Fermentation from FG to BG alters the levels of numerous endogenous substances [3,4]. Previously, Molina-Calle et al. reported that the levels of amino acids l-Arg, l-Asn, and l-Lys decreased after 36 days of heating FG, based on a metabolomics study [4]. Additionally, the levels of l-Asp, l-Ala, and l-Phe increased. The previous data regarding the changes in the free amino acids [4] are consistent with the data obtained in this study (Figure 2). However, previous studies lack data on the free d-amino acids in garlic samples. In this study, by using LC–MS/MS, we found only d-Ala in the FG samples (Figure 3).

Therefore, FG may contain d-Ala because it is present in grated raw and freeze-dried garlic samples. By contrast, the fermented BG foodstuff contains 12 kinds of free d-amino acids (Figure 3), with contents in the range of approximately 2–22% of those of the corresponding total (d- + l-) amino acids (Table 2). Because the other two garlic foodstuffs, which are not fermented, contain only d-Ala, most free d-amino acids occurring in BG may be produced during fermentation (steaming for approximately 14–30 days). The concentration of d-Ala in BG was approximately four-fold higher than those in the grated raw and freeze-dried garlic samples. Similarly, the concentration of l-Ala was approximately two- to three-fold higher than those in the grated raw and freeze-dried garlic samples.

During fermentation with heating under humid conditions, metabolic pathways involving amino acids could be formed. Consequently, several free l-amino acid levels were altered: the l-Arg, l-Asn, and l-Lys levels decreased, whereas the l-Asp, l-Ala, and l-Phe levels increased. Possibly accompanying these metabolic pathways, 12 kinds of free d-amino acids, such as d-Asn, d-Ala, d-Ser, d-Thr, d-Glu, d-Asp, d-Pro, d-Arg, d-Phe, d-Orn, d-Lys, and d-Tyr, may be produced in garlic via fermentation. The racemization of free l-amino acids to produce the corresponding d-amino acids is likely to occur. In addition, the thermal denaturation of proteins or peptides may cause the racemization of l-amino acids to d-amino acids. The l-Arg and l-Asn concentrations in BG were lower than those in FG, whereas the d-Arg and d-Asn levels were the highest in BG. However, for d-Ser, d-Asp, d-Ala, and d-Tyr, the concentrations of the corresponding l-amino acids in BG were also higher than those in FG. Therefore, at present, it is difficult to conclude that the production of free d-amino acids is caused by the racemization of free l-amino acids. Furthermore, the possibility that unknown metabolic pathways in garlic, which produce the free d-amino acids, are activated via fermentation, may not be negligible. Nevertheless, the d-amino acid contents in BG can be controlled during preparation by regulating the temperature, humidity, and duration of fermentation.

Although the nutritional effects of these free d-amino acids remain unclear, several papers have reported that d-amino acids exhibit physiological functions in mammals. Sasabe et al. have previously reported that numerous d-amino acids occur in the small intestine and that the co-produced hydrogen peroxide (H_2_O_2_) may protect the mucosal surface from pathogens via the d-amino acid-induced d-amino acid oxidase pathway [32]. Therefore, the ingestion of BG, which contains several free forms of d-amino acids, may aid in maintaining the normal functioning of the intestinal tract.

Among the free d-amino acids found in BG, d-Ser, which occurs at a relatively high percentage in BG foodstuffs, acts as a co-agonist of the NMDA receptor and has received attention in research on the central nervous system [33]. The intake of d-Ser with the second-generation antipsychotic risperidone or olanzapine was found to ameliorate negative symptoms in schizophrenia patients [34,35]. Previously, we reported significantly decreased levels of serum d-Ser in patients with schizophrenia [36,37]. Moreover, significantly increased levels of d-Ser were reported in the brains of laboratory rats that were systematically administered d-Ser in a microdialysis study [38,39]. Taken together, the results suggest that d-Ser-containing BG may be suitable for use as a daily d-Ser supplement.

d-Asp is also an endogenous free d-amino acid [40,41] and may act as a neurotransmitter for the NMDA receptor [42]. In addition, d-Asp may be involved in the biosynthesis of testosterone because blood testosterone levels increased in rats after intraperitoneal injection [43]. The biochemical mechanisms of d-Asp-functions in Leydig cells and spermatogonia have been postulated [41,44].

On the other hand, d-Trp, which has been reported to cause a loss of body weight gain in rats [11], was not found in BG.

Finally, because of the occurrence of several kinds of free d-amino acids in BG, its efficacy may not be identical to that of FG. Therefore, the pharmacological effects of BG should be analyzed by considering its levels of bioactive free d-amino acids.

## 3. Materials and Methods

### 3.1. Chemicals

Details of the reagents used in this study are presented in Supplementary Materials. CIMa-OSu was synthesized in our laboratory using a previously reported method [39].

### 3.2. Derivatization Procedure

Ten microliters of the sample was mixed with an internal standard (IS) mixture (Supplementary Materials) (10 μL of the prepared solution, according to the protocol included in the packaging of the reagent) and vortexed for 1 min. H_2_O (10 μL) was added to the mixture, which was then vortexed for 1 min and subsequently added with 20 mM (*R*)-CIMa-OSu in CH_3_CN (10 μL) and 30 mM DMAP in CH_3_CN (10 μL). The solution was vortexed vigorously for 1 min, after which the reaction was allowed to proceed at room temperature (22 °C) for 15 min. Subsequently, 0.1% formic acid in CH_3_CN (1.0 mL) was added to the solution, and the resultant solution was subjected to solid-phase extraction (SPE) using an SPE cartridge, InertSep^®^ NH_2_ (GL Sciences Inc., Tokyo, Japan), as described in our previous study for the miso sample [39]. The eluate (100 μL) was mixed with the mobile phase A/B (9/1, *v*/*v*, 100 μL) and filtered using Millex^®^-LG filters (0.20 μm). The filtrate was analyzed by LC–MS/MS.

### 3.3. Preparation of Garlic Samples

In this study, three types of commercial garlic foodstuffs, namely grated raw garlic, freeze-dried garlic, and fermented BG, were used. Two kinds of products belonging to each of these categories were evaluated, namely, grated raw garlic (*a*, *b*), freeze-dried garlic (*c*, *d*), and fermented BG (*e*, *f*). Of the six products, four were purchased from a local market in Chiba, Japan; one of the fermented BG products was kindly donated by BSM Agri Chiba (Chiba, Japan); and the other product was purchased online.

Aliquots of the two types of garlic samples (grated raw garlic and fermented BG) were added to 2.0 mL tubes, weighed, and extracted with methanol (MeOH) (1.0 mL/0.1 g garlic foodstuff) at 60 °C for 15 min in a PERSONAL-11 incubator (Taitec, Koshigaya, Japan) with gentle shaking. Subsequently, the samples were centrifuged twice (4 °C, 3000× *g* and 13,200× *g* for 15 min each), and the obtained supernatant was diluted 100-fold with distilled H_2_O and vigorously suspended. Ten microliters of the suspension was then filled in a brown 1.5 mL plastic tube, and the subsequent procedure was the same as that described in Section 3.2.

To obtain freeze-dried garlic, several chips were placed in a plastic zipper bag and pounded into a powder using a mallet. The powdered sample was extracted with MeOH (1.0 mL/0.1 g powder) and subjected to the same procedure as that described above.

### 3.4. LC–MS/MS

A triple quadrupole mass spectrometer, LCMS-8040 (Shimadzu, Kyoto, Japan), attached to an electrospray ionization interface was used in LC–MS/MS. Two pumps (LC-20AD), an autosampler (SIL-20AC) and column oven (CTO-20A), and PC software (LabSolutions ver. 5.80) (Shimadzu, Kyoto) were used. The temperature of the autosampler tray was set to 4 °C, and the analytical column was a Scherzo SS-C18^®^ (250 × 2.0 mm, 3 μm) (Imtakt Corporation, Kyoto, Japan) column that was constantly maintained at 60 °C in the column oven. The mobile phase comprised CH_3_CN and a pH-buffered solution of H_2_O/MeOH/10 mM ammonium formate (AcONH_4_) in H_2_O (pH 2.8) (5/2/3, *v*/*v*/*v*) (A) and 10 mM AcONH_4_ in [H_2_O/MeOH (3/7, *v*/*v*)] (B). The mobile phase was pumped constantly at a flow rate of 0.2 mL/min using the following (time) elution program: (0–20 min) B% = 10; (20.01–56 min) B% = 10–59; (56.01–60 min) B% = 59–100; (60.01–75 min) B% = 100; (75.01–90 min) B% = 10. The injection volume was 3.0 μL. The desolvation line and heat-block temperatures were adjusted to 250 °C and 400 °C, respectively. The nebulizer and drying gas flow rates, ion-spray voltage, and collision-induced dissociation gas pressure were the same as those in our previous report [39], namely, 3.0 and 10 L min^−1^, 4.5 kV, and 230 kPa, respectively. Ions were detected using the multiple reaction monitoring mode ([M + H]^+^ > 91.1) and quantified using MS/MS detection in the positive ion mode (Table S1). The 6-point calibration curves for each amino acid were prepared by plotting the ratio of the peak area to IS against the amino acid concentrations (l-Arg: 12.5–400 μM; other amino acids: 0.0125–50 μM, *n* = 4), which were set according to individual amino acid levels in the MeOH extract of the garlic foodstuffs. The accuracy and precision of the present LC–MS/MS method for the determination of free d- and l-amino acids were evaluated by spiking standard solutions (50.0, 200 μM for l-Arg and 6.25, 25 μM for other d- and l-amino acids) (*n* = 4). The LODs were calculated at signal to noise ratio 3 (*S*/*N* = 3).

## 4. Conclusions

This study revealed that several kinds of free d-amino acids were formed in fermented BG, but not in FG foodstuffs. LC–MS/MS with pre-column derivatization using CIMa-OSu could provide data regarding the contents of not only the free form of l- but also those of d-amino acids in garlic foodstuffs.

## Figures and Tables

**Table 1:** Resolutions (*R*_s_) of the amino acid enantiomers derivatized using CIMa-OSu and the mixed-mode Scherzo SS-C18^®^ column (250 × 2.0 mm, 3 μm).

Amino Acid	*R*_s_	Amino Acid	*R*_s_	Amino Acid	*R*_s_
Asn	2.48	Asp	2.67	Ile	15.52
Ala	9.23	His	4.14	Leu	16.05
Cit	3.33	Pro	1.94	Trp	11.18
Gln	2.55	Val	16.94	Phe	13.70
Ser	4.42	Met	13.14	Orn	5.51
Thr	7.98	Arg	2.99	Lys	5.00
Glu	3.95	KYN	8.58	Tyr	4.55

**Table 2:** Percentages (%) of free d-amino acids relative to the corresponding total amino acid content in commercial garlic foodstuffs *a*–*f*.

	Asn	Ala	Ser	Thr	Glu	Asp	Pro	Arg	Phe	Orn	Lys	Tyr
*a*	-	5.43	-	-	-	-	-	-	-	-	-	-
*b*	-	5.88	-	-	-	-	-	-	-	-	-	-
*c*	-	6.06	-	-	-	-	-	-	-	-	-	-
*d*	-	3.44	-	-	-	-	-	-	-	-	-	-
*e*	19.7	13.4	22.3	-	11.7	20.2	6.1	4.8	8.2	6.4	3.2	1.8
*f*	11.2	14.3	20.8	17.0	8.7	15.1	7.4	4.8	10.1	6.8	2.7	2.2

-: d-amino acid was not detected.

## Data Availability

The research data are not shared.

## Supplementary Materials

The following supporting information can be downloaded at https://www.mdpi.com/article/10.3390/molecules28041773/s1, Figure S1: Chromatograms of dl-Ser and GABA obtained using mobile phase A with different pH levels, Table S1: Transitions for multiple-reaction monitoring (MRM) of amino acids and the corresponding internal standard (IS), Table S2: Limit of detection (LOD, *S*/*N* = 3) for amino acids (fmol/injection), Table S3: Intra- and inter-day accuracy and precision of the proposed LC–MS/MS method for the determination of free d- and l-amino acids in garlic foodstuffs.
