# Supplementary material for: Determination of d- and l-Amino Acids in Garlic Foodstuffs by Liquid Chromatography–Tandem Mass Spectrometry
Source: Molecules. 2023 Feb 13;28(4):1773. doi: 10.3390/molecules28041773 (PMC9965777; doi:10.3390/molecules28041773)
Supplement: Supplementary file 1 [file molecules-28-01773-s001.zip › molecules-2185615-supplementary.pdf]

Molecules

Supplementary Materials

Determination of D- and L-Amino Acids in Garlic Foodstuffs by Liquid Chromatography–Tandem Mass Spectrometry

Mayu Onozato, Haruna Nakanoue, Tatsuya Sakamoto, Maho Umino, and Takeshi Fukushima *

Faculty of Pharmaceutical Sciences, Toho University; 2-2-1 Miyama, Funabashi-shi, Chiba 274-8510, Japan.

* Correspondence e-mail: t-fukushima@phar.toho-u.ac.jp

Contents

Chemical and reagents ……. Page 3

Figure S1 Chromatograms of DL-Ser and GABA obtained using mobile phase A at different pH levels. ……. Page 5

Table S1 Transitions for multiple-reaction monitoring (MRM) of amino acids and the corresponding internal standard (IS). ……. Page 6

Table S2 Limit of detection (LOD, *S*/*N* = 3) for amino acids (fmol/injection). ……. Page 7

Table S3 Intra- and inter-day accuracy and precision of the proposed LC–MS/MS method for the determination of free D- and L-amino acids in garlic foodstuffs. ……. Page 8

1 Chemicals and reagents

L-Alanine (Ala), L-arginine (Arg), L-asparagine (Asn), L-aspartic acid (Asp), L-citrulline (Cit), γ-aminobutyric acid (GABA), L-glutamine (Gln), L-glutamate (Glu), glycine (Gly), L-histidine (His), L-isoleucine (Ile), L-leucine (Leu), L-lysine (Lys), L-methionine (Met), L-phenylalanine (Phe), L-proline (Pro), L-serine (Ser), L-tryptophan (Trp), L-threonine (Thr), L-tyrosine (Tyr), L-valine (Val), and L-ornithine (Orn) were obtained from Kyowa Hakko Bio Co., Ltd. (Tokyo, Japan). D-Ala, D-Phe, D-Trp, D-Ser, LC–MS-grade CH_3OH, HPLC-grade formic acid, and APDSTAG® Wako Amino Acids Internal Standard Mixture Solution were obtained from FUJIFILM Wako Pure Chemical Corporation (Osaka, Japan). *N*,*N*-Dimethylaminopyridine (DMAP), D-Ala, D-Arg, D-Asn, D-Asp, D-Gln, D-Glu, D-His, D-Ile, D-Leu, D-Lys, D-Thr, and D-Val were purchased from Tokyo Chemical Industry Co., Ltd. (Tokyo, Japan), and D-kynurenine (KYN), L-KYN, D-Met, D-Tyr, DL-Orn, D-Pro, and ammonium formate were procured from Sigma-Aldrich Co., Ltd. (St. Louis, MO, USA). DL-Cit was purchased from Matrix Scientific (Columbia, SC, USA), and LC–MS-grade CH_3CN was obtained from Kanto Kagaku Co., Ltd. (Tokyo, Japan). The anion-exchange syringe-type cartridge, InertSep® NH_2 (50 mg/mL), was purchased from GL Sciences Inc. (Tokyo, Japan), and the water used was purified using a Milli-Q Labo system (Nihon Millipore Co. Ltd., Tokyo, Japan). Millex®-

LG filters (0.20 μm) were purchased from Merck Ltd. (Darmstadt, Germany).

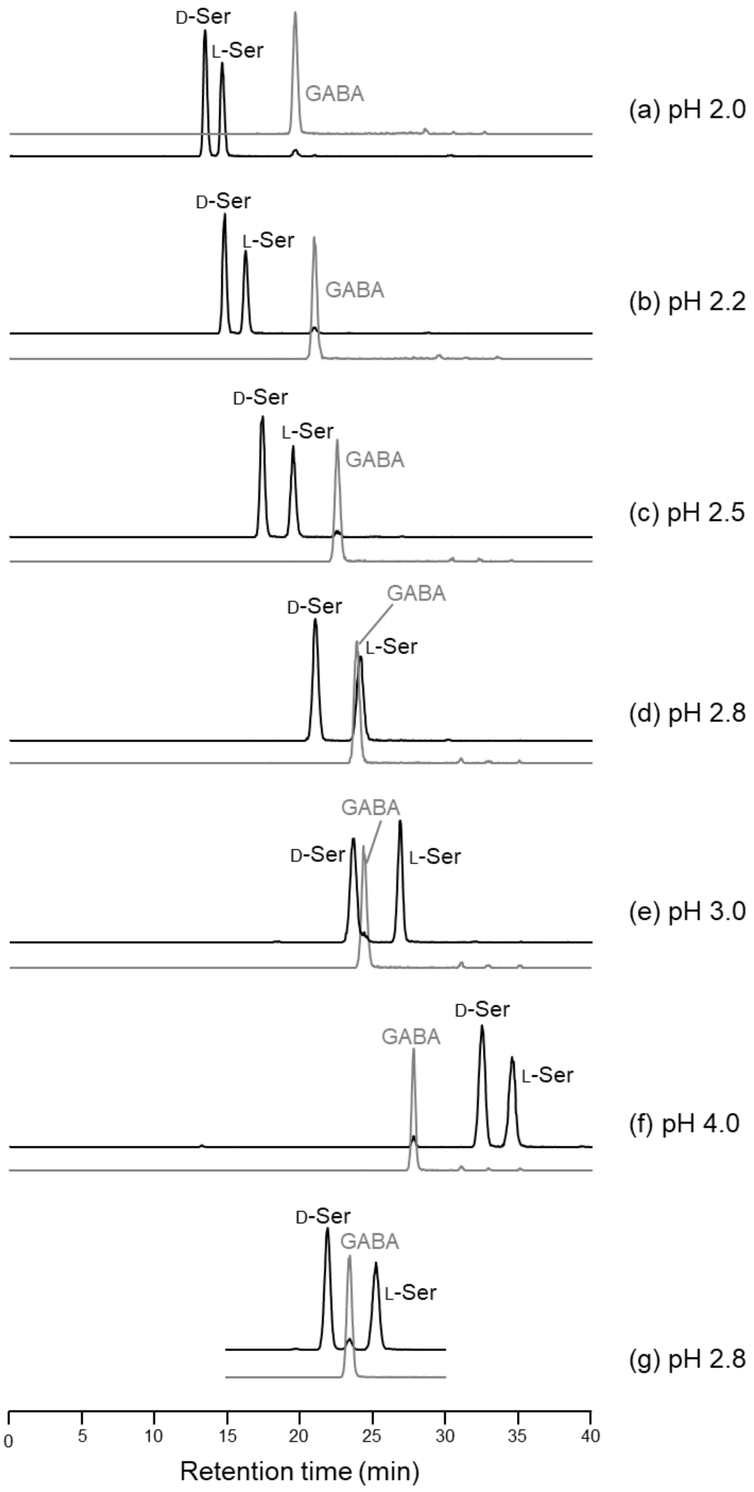

Figure S1 Chromatograms of DL-Ser and GABA obtained using mobile phase A at different pH levels. Mobile phase A) (a–f): H_2O/MeOH/10 mM ammonium formate (1/1/3, *v*/*v*/*v*), (g) H_2O/MeOH/10 mM ammonium formate (pH: 2.8, (5/2/3, *v*/*v*/*v*), B) 10 mM ammonium formate in [H_2O/MeOH (3/7, *v*/*v*)]. The time program for gradient elution of A) and B) is described in the text.

Table S1 Transitions for multiple-reaction monitoring (MRM) of amino acids and the corresponding internal standard (IS)

	Amino acid				IS		
	Precursor		Product		Precursor		Product
Asn	407.30	>	91.10	Asn-IS	413.30	>	91.10
Ala	364.20	>	91.10	Ala-IS	367.20	>	91.10
Cit	450.10	>	91.10	Cit-IS	454.10	>	91.10
Gln	421.20	>	91.10	Gln-IS	428.20	>	91.10
Ser	380.10	>	91.10	Ser-IS	384.10	>	91.10
Gly	350.20	>	91.10	Gly-IS	353.20	>	91.10
GABA	378.05	>	91.15	GABA		—	
Thr	394.10	>	91.10	Thr-IS	398.10	>	91.10
Glu	422.10	>	91.10	Glu-IS	428.10	>	91.10
Asp	408.10	>	91.10	Asp-IS	411.10	>	91.10
His	430.20	>	91.10	His-IS	439.20	>	91.10
Pro	390.10	>	91.10	Pro-IS	396.10	>	91.10
Val	392.10	>	91.10	Val-IS	398.10	>	91.10
Met	424.10	>	91.10	Met-IS	430.10	>	91.10
Arg	449.20	>	91.10	Arg-IS	453.20	>	91.10
KYN	483.30	>	91.10	KYN-IS		—	
Ile	406.10	>	91.10	Ile-IS	413.10	>	91.10
Leu	406.10	>	91.10	Leu-IS	409.10	>	91.10
Trp	479.30	>	91.10	Trp-IS	492.10	>	91.10
Phe	440.10	>	91.10	Phe-IS	450.10	>	91.10
Orn	681.40	>	91.10	Orn-IS	686.10	>	91.10
Lys	695.40	>	91.10	Lys-IS	703.10	>	91.10
Tyr	730.20	>	91.10	Tyr-IS	736.20	>	91.10

GABA-IS and KYN-IS were not included in APDSTAG® Wako Amino Acids Internal Standard Mixture Solution. Thus, Gly-IS and Met-IS were used as IS for GABA and KYN, respectively.

Table S2 Limit of detection (LOD, *S*/*N* = 3) for amino acids (fmol/injection).

	D-Amino acid		L-Amino acid	
	Previous study	This study	Previous study	This study
Asn	—	4.47	10.5	4.56
Ala	17.4	3.28	3.83	5.40
Cit	—	3.90	—	3.30
Gln	—	4.38	238	3.71
Ser	5.75	3.30	9.68	3.43
Thr	—	3.29	1.54	4.20
Glu	—	4.89	36.0	7.42
Asp	—	10.4	17.8	13.7
His	—	5.35	8.31	3.79
Pro	—	4.85	22.4	3.30
Val	—	2.93	2.12	4.28
Met	—	3.11	1.8	3.87
Arg	—	2.65	4.52	2.42
KYN	—	3.10	—	3.38
Ile	—	3.48	0.501	4.07
Leu	—	2.94	0.628	3.87
Trp	—	2.49	14.4	2.68
Phe	—	3.23	4.20	2.94
Orn	—	2.41	2.39	2.77
Lys	—	2.41	4.07	2.55
Tyr	—	2.07	4.25	2.66

	Previous study	This study
GABA	—	5.04
Gly	12.6	3.27
β-Ala	—	3.92

Table S3 Intra- and inter-day accuracy and precision of the proposed LC-MS/MS for the determination of free D- and L-amino acid in garlic foodstuff.

		Intra-day		Inter-day				Intra-day		Inter-day				Intra-day		Inter-day	
		Accuracy	Precision	Accuracy	Precision			Accuracy	Precision	Accuracy	Precision			Accuracy	Precision	Accuracy	Precision
Amino acids	Amount spiked (μM)	Recovery%	RSD%	Recovery%	RSD%	Amino acids	Amount spiked (μM)	Recovery%	RSD%	Recovery%	RSD%	Amino acids	Amount spiked (μM)	Recovery%	RSD%	Recovery%	RSD%
D-Asn	6.25	101	2.57	98.6	4.11	D-Glu	6.25	80.2	6.24	75.3	4.54	L-KYN	6.25	102	3.42	123	9.19
	25	97.8	2.46	96.5	2.24		25	81.8	1.48	93.0	3.15		25	99.8	8.56	127	2.95
L-Asn	6.25	99.8	1.59	97.1	1.20	L-Glu	6.25	101	3.48	84.4	2.47	D-Ile	6.25	101	1.97	88.8	1.75
	25	97.2	1.72	94.4	2.41		25	82.0	2.10	74.4	2.71		25	97.2	2.76	85.4	1.46
D-Ala	6.25	78.8	2.01	87.3	2.52	D-Asp	6.25	83.3	8.53	74.0	5.97	L-Ile	6.25	98.3	2.20	91.2	0.83
	25	76.4	1.76	82.3	2.47		25	83.2	3.67	107	1.86		25	100	0.33	88.1	3.03
L-Ala	6.25	97.2	1.76	92.6	0.85	L-Asp	6.25	90.6	6.70	82.5	1.42	D-Leu	6.25	98.2	1.70	87.3	1.65
	25	89.6	2.63	84.8	1.28		25	81.2	3.41	70.3	3.00		25	94.7	2.17	83.7	1.36
β-Ala	6.25	72.3	2.31	84.6	1.28	D-His	6.25	108	2.87	96.4	4.20	L-Leu	6.25	95.6	2.39	86.1	1.59
	25	78.8	2.60	84.9	0.98		25	112	3.71	96.2	3.28		25	94.4	1.59	83.3	0.85
D-Cit	6.25	111	7.58	106	2.18	L-His	6.25	116	1.49	103	3.17	D-Trp	6.25	101	2.83	90.8	3.39
	25	119	4.34	102	0.56		25	117	3.59	103	1.69		25	100	1.25	91.0	1.18
L-Cit	6.25	93.4	2.36	88.6	1.03	D-Pro	6.25	95.1	2.98	78.5	1.20	L-Trp	6.25	100	3.18	103	4.10
	25	96.5	7.47	85.4	1.09		25	93.7	1.04	78.7	2.16		25	99.8	0.89	102	3.96
D-Gln	6.25	101	3.42	110	6.53	L-Pro	6.25	93.7	1.72	83.7	0.91	D-Phe	6.25	97.8	0.61	85.8	0.69
	25	99.6	1.73	109	1.96		25	88.5	0.69	76.8	2.42		25	97.1	2.09	81.7	0.60
L-Gln	6.25	88.8	5.12	109	6.81	D-Val	6.25	115	3.84	103	3.07	L-Phe	6.25	102	1.46	94.5	0.94
	25	96.6	2.93	105	4.04		25	102	6.80	99.3	1.56		25	104	0.25	91.6	0.79
D-Ser	6.25	86.0	7.10	79.8	3.55	L-Val	6.25	111	1.37	98.2	3.34	D-Orn	6.25	62.5	0.77	106	1.59
	25	83.2	3.32	76.3	2.54		25	105	3.06	99.8	2.20		25	65.4	3.18	105	3.50
L-Ser	6.25	91.0	5.50	84.5	1.66	D-Met	6.25	116	4.08	99.5	9.97	L-Orn	6.25	64.6	1.79	105	3.43
	25	80.1	1.90	74.4	1.13		25	119	1.23	104	1.89		25	64.5	3.12	102	3.07
Gly	6.25	100	2.24	104	1.77	L-Met	6.25	113	5.58	101	3.88	D-Lys	6.25	63.3	3.32	104	1.34
	25	99.3	2.13	100	2.41		25	109	3.12	102	1.44		25	63.9	1.10	105	4.16
GABA	6.25	122	5.39	106	3.50	D-Arg	6.25	92.8	2.06	105	2.26	L-Lys	6.25	77.2	1.40	101	1.78
	25	113	6.00	100	2.42		25	89.8	2.04	102	4.14		25	68.3	0.94	100	2.95
D-Thr	6.25	92.3	2.46	84.4	5.22	L-Arg	50	94.0	0.94	107	2.29	D-Tyr	6.25	99.7	2.50	97.1	2.03
	25	84.9	1.65	78.8	2.65		200	89.5	1.51	100	1.63		25	101	1.26	92.2	1.73
L-Thr	6.25	89.7	1.41	83.7	2.63	D-KYN	6.25	101	6.04	116	9.08	L-Tyr	6.25	103	0.60	103	2.07
	25	84.9	2.04	75.1	1.93		25	99.9	3.67	115	3.58		25	111	1.31	104	2.37
